# Prevalence of frailty and its association with cognition in preclinical Alzheimer’s disease: a cross-sectional analysis of baseline data from the A4 study

**DOI:** 10.1093/ageing/afaf378

**Published:** 2026-01-22

**Authors:** Andrew L H Huynh, Shunran Wang, Kathryn Lee, Sanka Amadoru, Scott Wrigley, Georgios Zisis, Karin Ernstrom, Rema Raman, Paul Aisen, Reisa A Sperling, Colin L Masters, David Ward, Paul A Yates

**Affiliations:** Department of Geriatric Medicine, Austin Health, Heidelberg, Melbourne, Victoria, Australia; Department of Medicine, Austin Health, The University of Melbourne, Heidelberg, Melbourne, Victoria, Australia; The Florey Institute of Neuroscience and Mental Health, Parkville, Melbourne, Victoria, Australia; Alzheimer’s Therapeutic Research Institute, Keck School of Medicine, University of Southern California, San Diego, CA, USA; Department of Geriatric Medicine, Austin Health, Heidelberg, Melbourne, Victoria, Australia; Department of Geriatric Medicine, Austin Health, Heidelberg, Melbourne, Victoria, Australia; Department of Medicine, Austin Health, The University of Melbourne, Heidelberg, Melbourne, Victoria, Australia; Department of Geriatric Medicine, Austin Health, Heidelberg, Melbourne, Victoria, Australia; The Florey Institute of Neuroscience and Mental Health, Parkville, Melbourne, Victoria, Australia; Alzheimer’s Therapeutic Research Institute, Keck School of Medicine, University of Southern California, San Diego, CA, USA; Alzheimer’s Therapeutic Research Institute, Keck School of Medicine, University of Southern California, San Diego, CA, USA; Alzheimer’s Therapeutic Research Institute, Keck School of Medicine, University of Southern California, San Diego, CA, USA; Department of Neurology, Massachusetts General Hospital, Boston, MA, USA; Center for Alzheimer Research and Treatment, Department of Neurology, Brigham and Women's Hospital, Boston, MA, USA; Harvard Medical School, Boston, MA, USA; The Florey Institute of Neuroscience and Mental Health, The University of Melbourne, Parkville, Melbourne, Victoria, Australia; Centre for Health Services Research, Faculty of Health, Medicine and Behavioural Sciences, The University of Queensland, Princess Alexandra Hospital, Woolloongabba, Queensland, Australia; Australian Frailty Network, The University of Queensland, Woolloongabba, Queensland, Australia; Department of Geriatric Medicine, Austin Health, Heidelberg, Melbourne, Victoria, Australia; Department of Medicine, Austin Health, The University of Melbourne, Heidelberg, Melbourne, Victoria, Australia

**Keywords:** frailty, Alzheimer’s disease, amyloid, cognitive decline, older adults

## Abstract

**Background:**

The prevalence and role of frailty in preclinical Alzheimer’s disease (AD) is unclear.

**Methods:**

Cross-sectional analyses of pre-randomization data from the Anti-Amyloid Treatment in Asymptomatic AD (A4) study were analysed to derive two models of a frailty index (FI)—full [FI-Full] and cognitive variables removed [FI-CVR]. The prevalence of frailty (FI > 0.25) according to amyloid status (Aβ+/−), and the association of frailty and cognition (determined by the Preclinical Alzheimer Cognitive Composite (PACC) score) and whether frailty moderates the relationship between amyloid status and cognition was assessed, adjusting for age, sex and education.

**Results:**

Four thousand four hundred eighty-six participants were included (mean age 71.3 ± 4.7 years, 30% participants Aβ+, 59% female). The prevalence of frailty in preclinical AD was 22% (or 44% when cognitive variables were removed from the FI). Using either FI model, in adjusted analyses, Aβ+ participants were more likely to be frail compared to Aβ− [FI-Full—Odds ratio (OR) 1.43 95% confidence interval (CI) 1.20–1.71, *P* < .001; FI-CVR—OR 1.21 95% CI 1.05–1.40, *P* < .008]. Frail participants had lower PACC scores compared to non-frail participants, on average (FI-Full—PACC score −0.58 95% CI −0.76 to −0.40, *P* < .001; FI-CVR—PACC score −0.26 95% CI −0.40 to −0.12, *P* < .001). Frailty did not influence the relationship between Aβ status and cognition.

**Conclusions:**

In a cohort screened for a preclinical AD trial, elevated Aβ levels were associated with frailty and frailty was associated with reduced cognitive performance independent of elevated Aβ levels. These associations, and whether or not frailty is associated with longitudinal cognitive decline independent of Aβ status, warrant further study.

## Key Points

- The prevalence of frailty in a clinical trial cohort preclinical AD is 22%.
- Elevated amyloid β (Aβ) levels were associated with frailty.
- Individuals with preclinical AD were 43% more likely to be frail.
- Frailty was associated with lower cognitive performance, independent of Aβ status.
- Frailty did not influence the relationship between Aβ status and cognition.

## Introduction

Frailty is a clinical syndrome characterised by a state of increased vulnerability to stressors resulting from a decline in physiological reserve in multiple systems [1, 2]. It is associated with an increased risk of adverse outcomes including falls, fractures, hospitalisation, long term residential care admission, mortality and increased healthcare costs [2–4].

Frailty can be described both as a phenotype using Fried’s criteria [3] or using a deficits accumulation model of age-related health deficits in a frailty index (FI) [5]. Frailty is an independent predictor of incident all-cause dementia [6, 7], and increases and accelerates prior to incident all-cause dementia [8]. Increased frailty in people who were cognitively unimpaired increased the risk of progression to mild cognitive impairment (MCI) and dementia, and in people who had MCI, increased frailty increased the risk of progression to dementia, independent of apolipoprotein E (APOE) ε4 carrier status [9].

In Alzheimer’s disease (AD), frailty has been shown to moderate the neuropathological features of AD and the clinical expression of dementia [10]. People with low levels of AD neuropathology were more likely to progress to dementia if they were frailer [10]. However, the prevalence of frailty in preclinical AD, where individuals are cognitively unimpaired but have elevated amyloid β (Aβ) burden [11], is unclear. The Anti-Amyloid Treatment in Asymptomatic AD (A4) study was a phase 3 clinical prevention trial (ClinicalTrials.gov Number: NCT02008357) to determine if solanezumab, a monoclonal antibody against monomeric forms of Aβ, could slow the progression of cognitive decline in people with preclinical AD. Whilst frailty is a common [12] and potentially modifiable risk factor for dementia, it is not usually measured in clinical drug trials for AD [13].

We aimed to (i) compare the prevalence of frailty between individuals with and without preclinical AD as determined by amyloid positron emission tomography (PET), and (ii) in a cognitively unimpaired cohort with amyloid PET biomarker measurement, to examine the association of frailty (independent variable) and cognition (dependent variable) and whether frailty moderates the relationship between Aβ status and cognition.

## Methods

### Study design and participants

Cross-sectional analysis of participants was conducted amongst participants who underwent screening and had an amyloid PET (pre-randomization cohort) in the A4 study. The A4 protocol has been previously reported [14, 15], with relevant information for this study described below.

Participants were screened for the A4 study from 67 sites across Australia, Canada, Japan and the USA between April 2014 to December 2017. Eligibility for screening were if participants were aged 65–85 years old, cognitively unimpaired with a global Cognitive Dementia Rating (CDR) [16] of 0 (range 0–3, with 0 representing no cognitive impairment and 3 indicating severe dementia), Mini-Mental State Examination (MMSE) [17] score of 25–30 (range 0–30, with lower scores representing poorer cognition), a Wechsler Memory Scale Logical Memory Delayed Recall (LMDR IIa) [18, 19] score of 6–18 (range 0–25, with lower scores representing fewer details recalled), and had a study partner who was familiar with the participant’s cognition and activities of daily living. Key exclusion criteria included a diagnosis of MCI or dementia, and serious or unstable medical conditions, although participants with well-controlled treated hypertension, diabetes mellitus, dyslipidaemia and other medical conditions, and mild-to-moderate small vessel cerebrovascular disease were eligible for screening.

At screening, demographics collected included age in years, education in years, sex, race, ethnicity, marital status, medical co-morbidities, a family history of dementia. Biomarkers collected included APOE ε4 carrier status and elevated amyloid levels (Aβ+). Measurements of Aβ+, cognition and frailty are described below.

### Amyloid positron emission tomography imaging

Participants who were eligible for screening underwent amyloid PET imaging, acquired 50–70 minutes post-administration of ^18^F-florbetapir. Brain amyloid levels were assessed using the mean cortical standardised uptake value ratio (SUVR) with a cerebellar reference region [20]. Amyloid status (Aβ+/−) for each participant was determined by an algorithm that combined quantitative SUVR methods and qualitative visual readings done at a central laboratory. Aβ+ was defined as a quantitative SUVR greater of 1.15 [20]. An SUVR between 1.10 and 1.15 was considered Aβ+ only when a visual read was also considered positive by two-reader consensus [14].

### Cognitive testing

Participants’ cognition was evaluated via the Preclinical Alzheimer’s Cognitive Composite (PACC), a validated score that assesses amyloid-related cognitive decline in cognitively unimpaired participants [21–23]. The PACC comprises four components: MMSE [17] score, LMDR IIa [18, 19], the Free and Cued Selective Reminding Test (FCSRT) [24] score (range 0–96, with lower scores representing increased memory impairment), and the Digital Symbol Substitution Test from the Wechsler Adult Intelligence Scale-Revised [25]. Each of the four components is converted to a standardised *z*-score by subtracting the baseline mean for that component and diving by the baseline standard deviation for that component. The PACC score is the sum of the four component z-scores. Higher *z*-scores represent better cognitive performance.

### Frailty

Frailty of participants was measured using an FI. The FI represents a deficits accumulation model of frailty [5], defined as a ratio of the sum of a participant’s health deficits present to the total number of health deficits measured (range 0–1, where a higher FI score denoted a higher degree of frailty). Health deficits included in the composition of FIs are those that are associated with health (symptoms, signs, disabilities and diseases), increase in prevalence with advancing age, are present in more than 1% of respondents, and do not saturate too early (i.e. not common before the age of 65 years old) [26]. The FI variables selected for the FI were collected from the A4 screening questionnaires and physical and neurological examination. We derived two FI models—the FI-Full and the FI-cognitive variables removed (CVR). FI-Full included all 51 eligible variables in its calculation, whilst FI-CVR included only 36 variables following the exclusion of 15 variables that were deemed possibly confounding in the relationship between frailty and cognition (i.e. potential indicators of early decline in cognition or markers of neurological dysfunction). The variables used in both models are shown in Appendix Table 1 in the Supplementary Data section, with excluded variables in FI-CVR highlighted with an asterisk (*). Scoring and prevalence of variables for the FI are shown in Appendix Table 2 in the Supplementary Data section. Frailty was defined as a FI > 0.25, as has been previously published [27].

### Statistical analyses

Baseline demographics were summarised by frail and non-frail participants, with frequencies and percentages for categorical variables, and with mean and standard deviation for continuous data. Comparisons between groups were performed using *t*-tests for continuous variables, and Pearson’s chi-squared test (χ^2^) for categorical data. Multivariable logistic regression was used to investigate the difference in the prevalence of frailty between amyloid status (Aβ+/−) groups, adjusting for age, sex, education and APOE ε4 carrier status. Analysis of covariance (ANCOVA) models were used to examine the influence of frailty (either FI > 0.25 or using FI as a continuous variable) on the relationship between amyloid status and cognition (PACC score). There were no transformations applied to the FI in the models. The model included PACC as the outcome with main effects of frailty and amyloid status with frailty as the moderator, an interaction term of frailty and amyloid, adjusting for age, sex and education. If the interaction terms of frailty and amyloid was found to be statistically significant (a *P*-value of less than .05), then frailty would be considered influential on the relationship between PACC and amyloid group. All analyses were performed for both FI-Full and FI-CVR, separately, using R Version 4.4.1 (R Foundation for Statistical Computing).

## Results

### Baseline demographics

There were 4486 pre-randomization participants who were screened for the A4 study and underwent amyloid PET scanning. Characteristics of participants (total/frail/non-frail) are shown in Table 1. Of the total participants, the mean ± standard deviation (SD) age was 71.3 ± 4.7 years old, 2663 (59%) were female and the average education of participants was high (mean ± SD education 16.6 ± 2.8 years). The participants were predominantly Caucasian (*n* = 4093, 91%), married (*n* = 3166, 71%) and retired (*n* = 3401, 76%). In total, 1323 (30%) participants were categorised as Aβ+ on amyloid PET, 3113 (69%) participants reported a family history of dementia, and 1550 (35%) participants carried at least one APOE ε4 allele.

**Table 1:** Characteristics of participants who underwent screening amyloid PET for A4 study by frail/non-frail groups

	Total	FI-Full			FI-CVR		
	(*n* = 4486)	Frail (FI > 0.25) (*n* = 765)	Non-frail (FI ≤ 0.25) (*n* = 3721)	*P*-value	Frail (FI > 0.25) (*n* = 1718)	Non-frail (FI ≤ 0.25) (*n* = 2768)	*P*-value
Aβ+	1323 (30)	291 (38)	1032 (28)	<0.001	579 (34)	744 (27)	<.001
APOE ε4 carrier	1550 (35)	279 (37)	1271 (35)	0.184	627 (37)	923 (34)	.023
Age (years)	71.3 ± 4.7	72.7 ± 5.1	71.0 ± 4.5	<0.001	72.3 ± 4.9	70.7 ± 4.4	<.001
Education (years)	16.6 ± 2.8	16.3 ± 2.8	16.6 ± 2.8	0.002	16.5 ± 2.8	16.7 ± 2.9	.024
Female	2663 (59)	426 (56)	2237 (60)	0.023	965 (56)	1698 (61)	<.001
Race American Indian/Alaskan Native Asian Black/African American Multiple Native Hawaiian/Pacific Islander Caucasian Unknown/not reported	9 (0) 170 (4) 159 (4) 27 (1) 2 (0) 4093 (91) 26 (1)	3 (0) 21 (3) 17 (2) 7 (1) 1 (0) 711 (93) 5 (1)	6 (0) 149 (4) 142 (4) 20 (1) 1 (0) 3382 (91) 21 (1)	0.055	4 (0) 31 (2) 52 (3) 8 (1) 1 (0) 1614 (94) 8 (1)	5 (0) 139 (5) 107 (4) 19 (1) 1 (0) 2479 (90) 18 (1)	<.001
Ethnicity Hispanic or Latino Not Hispanic or Latino Unknown	142 (3) 4309 (96) 35 (1)	31 (4) 728 (95) 6 (1)	111 (3) 3581 (96) 29 (1)	0.306	62 (4) 1642 (96) 14 (1)	80 (3) 2667 (96) 21 (1)	.399
Marital status Married Widowed Divorced Never married Unknown/Other	3166 (71) 426 (10) 628 (14) 183 (4) 83 (2)	513 (67) 80 (11) 116 (15) 40 (5) 16 (2)	2653 (71) 346 (9) 512 (14) 143 (4) 67 (2)	0.154	1208 (70) 173 (10) 240 (14) 76 (4) 21 (1)	1958 (71) 253 (9) 388 (14) 107 (4) 62 (2)	.102
Retired	3401 (76)	593 (78)	2808 (76)	0.439	1337 (78)	2064 (75)	.003
Family history of dementia	3113 (69)	520 (68)	2593 (70)	0.349	1172 (68)	1941 (70)	.179
MMSE Digital symbol substitution test LMDR IIa FCSRT	28.8 ± 1.2 43.8 ± 9.0 11.7 ± 3.2 76.3 ± 5.9	28.6 ± 1.3 40.8 ± 8.9 11.4 ± 3.2 76.6 ± 5.9	28.9 ± 1.2 44.4 ± 8.9 11.8 ± 3.2 75.1 ± 6.1	<0.001 <0.001 0.005 <0.001	28.7 ± 1.2 42.0 ± 8.9 11.7 ± 3.2 75.7 ± 5.9	28.9 ± 1.2 44.8 ± 8.8 11.7 ± 3.2 76.7 ± 5.9	.001 <.001 .596 <.001
PACC (Sum of *z*-scores)	0 ± 2.5	−0.8 ± 2.6	0.2 ± 2.5	<0.001	−0.4 ± 2.6	0.2 ± 2.5	<.001

Data are presented as mean ± standard deviation, or number (percentage); FI = frailty index, CVR = cognitive variables removed, Aβ+ = elevated amyloid β, PACC = Preclinical Alzheimer’s Cognitive Composite, APOE = apolipoprotein E, MMSE = Mini-Mental State Examination score,

LMDR IIa = Welscher Memory Scale Logical Memory Delayed Recall, FCSRT = Free and Cued Selective Reminding Test.

### Prevalence of frailty

FI-Full scores in the A4 pre-randomization cohort ranged from 0 to 0.53 with a mean of 0.19 (SD 0.07), and for FI-CVR the range was 0 to 0.53 with a mean of 0.22 (SD 0.09). When the prevalence of frailty was determined using FI > 0.25, it was 17% [95% confidence interval (CI) 16%–18%] using FI-Full and 38% (95% CI 37%–40%) using FI-CVR. For both FI models, there remained a higher proportion of Aβ+ frail participants than Aβ+ non-frail participants (FI-Full—frail 38% 95% CI 35%–42%, non-frail 28% 95% CI 26%–29%, *P* < .001; FI-CVR—frail 34% 95% CI 32%–36%, non-frail 27% 95% CI 25%–29%, *P* < .001) (Table 2). Participants categorised as frail were older, had a higher proportion who were male, and had similar education in years compared to participants categorised as non-frail. Using FI-CVR, there was a statistical significance in APOE ε4 carrier status and retirement status between frail and non-frail participants (APOE ε4 carrier—frail 37%, non-frail 34%, *P* = .023; Retired—frail 78%, non-frail 75%, *P* = .003). The FI-Full model had similar proportions to FI-CVR comparing frail and non-frail participants in APOE ε4 carrier and retirement status, but there was no statistical significance (APOE ε4 carrier—frail 37%, non-frail 35%, *P* = .184; Retired—frail 78%, non-frail 76%, *P* = .439).

**Table 2:** Amyloid status by frailty

*(a) FI-Full model*			
	Frail (FI > 0.25) (${n}={765}$)	Non-frail (FI ≤ 0.25) (${n}={3721}$)	Total (${n}={4486}$)
Aβ+	291 (38 [35–42])	1032 (28 [26–29])	1323 (29 [28–31])
Aβ-	474 (62 [58–65])	2689 (72 [71–74])	3163 (71 [69–72])
*(b) FI-CVR*			
	Frail (FI > 0.25) (${n}={1718}$)	Non-frail (FI ≤ 0.25) (${n}={2768}$)	Total (${n}={4486}$)
Aβ+	579 (34 [32–36])	744 (27 [25–29])	1323 (29 [28–31])
Aβ-	1139 (66 [64–68])	2024 (73 [71–75])	3163 (71 [69–72])

Adjusted odds ratio for prevalence of frailty comparing Aβ+ to Aβ– 1.43 (95% CI 1.20–1.71), *P* < .001

Adjusted odds ratio for prevalence of frailty comparing Aβ+ to Aβ– 1.21 (95% CI 1.05–1.40), *P* < .001

Prevalence of frailty determined using multivariable logistic regression model adjusting for age, sex, education and APOE ε4 carrier status

Data are presented as number (percentage [95%CI]); FI = frailty index, CVR = cognitive variables removed.

### Frailty and amyloid status

**Figure 1:**
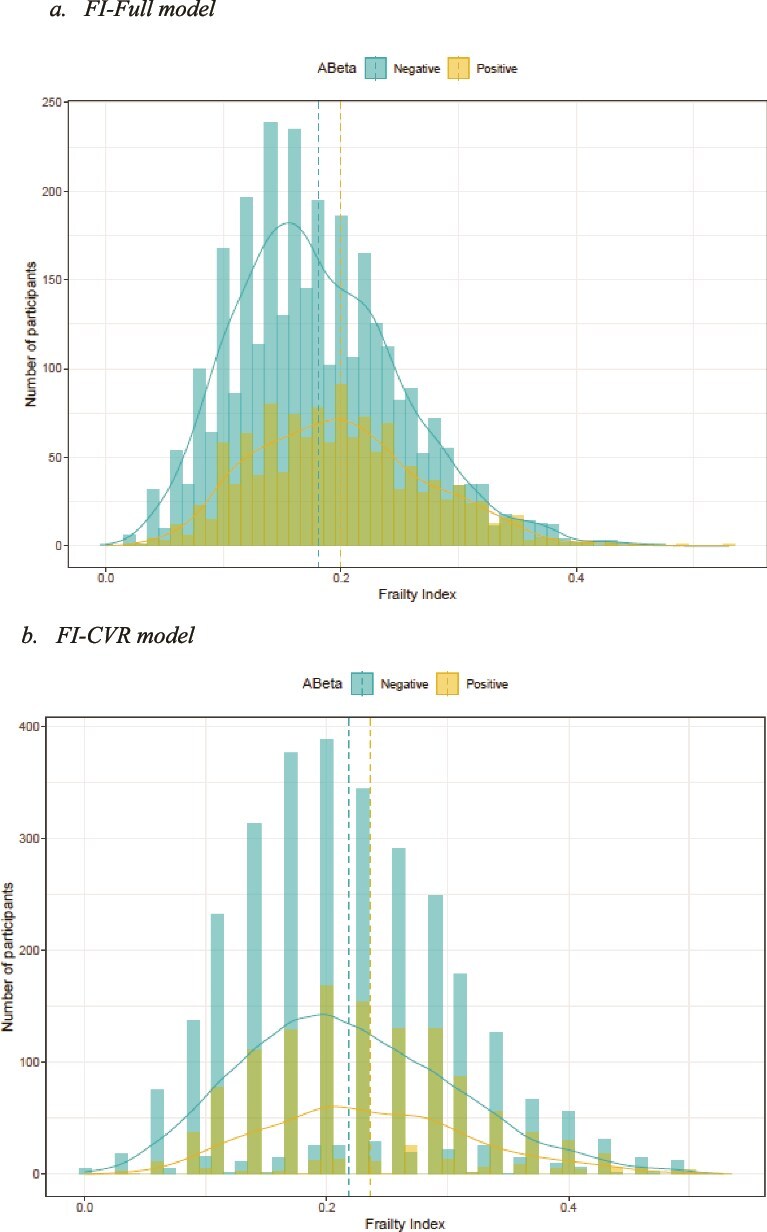
Frailty index by amyloid status (a) ‘FI-Full model’, (b) ‘FI-CVR model’ Dashed lines represent the mean.

FI scores for both models stratified by amyloid status are shown in Figure 1. There was a higher proportion of Aβ+ participants who were frail than Aβ– participants who were frail in both FI models (FI-Full—Aβ+ 22% 95% CI 20%–24%, Aβ– 15% 95% CI 14%–16%, *P* < .001; FI-CVR—Aβ+ 44% 95% CI 41%–46%, Aβ– 36% 95% CI 34%–38%, *P* < .001). In a multivariable logistic regression model, adjusting for age, sex, education and APOE ε4 carrier status, Aβ+ participants were more likely to be frail compared to Aβ– participants [FI-Full—Odds ratio (OR) 1.43 95% CI 1.20–1.71, *P* < .001; FI-CVR—OR 1.21 95% CI 1.05–1.40, *P* < .008].

### Frailty and cognition

The PACC score and its components according to frailty status are shown in Table 1. Whilst significant statistical differences were observed for some PACC components, the absolute differences were small. Given that the differences and variability in each PACC component between frail and non-frail groups were too small to draw conclusions, only the PACC (composite score) was used for regression analysis. The linear ANCOVA model using amyloid status and either frailty status or FI as a continuous variable as independent variables and PACC as the dependent variable with and without the interaction terms of amyloid and frailty status adjusted for age, sex and education are shown in Table 3 and Table 4, respectively. Frail participants had lower PACC scores on average compared to non-frail participants after adjusting for age, sex, education and amyloid status (FI-Full—PACC score −0.58 95% CI –0.76 to −0.40, *P* < .001; FI-CVR—PACC score −0.26 95% CI –0.40 to −0.12, *P* < .001), and Aβ+ participants had lower PACC scores on average compared to Aβ– participants (FI-Full—PACC score −0.39 95% CI –0.54 to −0.22, *P* < .001; FI-CVR—PACC score −0.41 95% CI –0.55 to −0.26, *P* < .001). Similar results were observed using FI as continuous variables, namely participants with a higher FI had on average a lower PACC score (FI-Full—PACC score −3.46 95% CI –4.41 to −2.51, *P* < .001; FI-CVR—PACC score −1.43 95% CI –2.21 to −0.66, *P* < .001). Thus, frailty status was associated with cognition regardless of amyloid status.

**Table 3:** Associations of frailty (FI > 0.25) and amyloid status with preclinical Alzheimer’s cognitive composite

*(a) FI-Full model*				
	Without interaction term		With interaction term	
	Estimate (95% CI)	*P*-value	Estimate (95% CI)	*P*-value
Frailty (FI > 0.25)	−0.58 (−0.76 to −0.40)	<.001	−0.56 (−0.78 to −0.33)	<.001
Aβ+	−0.39 (−0.54 to −0.24)	<.001	−0.38 (−0.54 to −0.21)	<.001
Interaction term FI > 0.25: Aβ+			−0.06 (−0.43–0.31)	.747
*(b) FI-CVR*				
	Without interaction term		With interaction term	
	Estimate (95% CI)	*P*-value	Estimate (95% CI)	*P*-value
Frailty (FI > 0.25)	−0.26 (−0.40 – −0.12)	<.001	−0.25 (−0.42 to −0.08)	<.001
Aβ+	−0.41 (−0.55—−0.26)	<.001	−0.39 (−0.58 to −0.19)	<.001
Interaction term FI > 0.25: Aβ+			−0.05 (−0.35–0.25)	.762

FI = frailty index, CVR = cognitive variables removed, CI = confidence interval, Aβ+ = elevated amyloid beta levels

Estimates were derived from multivariable linear regression models including covariates of age, sex and education.

**Table 4:** Associations of frailty (FI as a continuous variable) and amyloid status with preclinical Alzheimer’s cognitive composite

*(a) FI-Full model*				
	Without interaction term		With interaction term	
	Estimate (95% CI)	*P*-value	Estimate (95% CI)	*P*-value
FI	−3.46 (−4.41 to −2.51)	<.001	−3.46 (−4.59 to −2.33)	<.001
Aβ+	−0.37 (−0.51 to −0.22)	<.001	−0.37 (−0.79–0.05)	.084
Interaction term FI: Aβ+			0.01 (−2.01–2.04)	.989
*(b) FI-CVR*				
	Without interaction term		With interaction term	
	Estimate (95% CI)	*P*-value	Estimate (95% CI)	*P*-value
FI	−1.43 (−2.21 to −0.66)	<.001	−1.66 (−2.57 to −0.75)	<.001
Aβ+	−0.40 (−0.55 to −0.25)	<.001	−0.58 (−1.00 to −0.17)	.006
Interaction term FI: Aβ+			0.79 (−0.90–2.48)	.358

FI = frailty index, CVR = cognitive variables removed, CI = confidence interval, Aβ+ = elevated amyloid beta levels

Estimates were derived from multivariable linear regression models including covariates of age, sex and education.

## Discussion

In this cohort of participants screened for a preclinical AD trial, our study found that (i) the prevalence of frailty in preclinical AD was 22% (or 44% when cognitive variables were removed from the FI); (ii) Aβ+ participants were more likely to be frail compared to Aβ−; (iii) frailty was associated with lower cognitive performance compared to non-frail participants regardless of amyloid status; and (iv) frailty did not influence the relationship between Aβ status and cognition.

The prevalence of frailty in this community-dwelling cohort screened for the A4 study was 17% using FI-Full or 38% using FI-CVR. The 17% prevalence using FI-Full (51-items) is lower than the estimated global prevalence of frailty of 24% (95% CI 22%–26%), derived from a systematic review and meta-analyses from 62 countries [28]. The lower prevalence of frailty reported in the A4 pre-randomization cohort could be due to the inclusion criteria requiring participants to be aged 65–85 years old and excluded participants who had a diagnosis of cognitive impairment or unstable medical conditions. In comparison, the global study, which included adults aged 50 years old and above, had no exclusion criteria. In countries where the A4 study was conducted, the prevalence of frailty in community-dwelling older adults was 11% in Australia [29], 20% in Canada [30], 9% in Japan [31], and 15% in the USA [32]. In contrast, the 38% prevalence of frailty using FI-CVR, a 36-item FI that excluded variables in FI-Full that were potential indicators of early decline in cognition or markers of neurological is higher than the global prevalence. However, is important to note that our overall findings of the influence of frailty on the relationship between amyloid status and cognition were similar in both models.

To our knowledge, this is the first study to report the prevalence of frailty in preclinical AD. The Multidomain Alzheimer’s Preventive Trial (MAPT) was a prevention intervention study that had 269 community-dwelling participants from France and Monaco with a CDR 0–0.5 and who underwent an amyloid PET [33]. Using a 19-item FI that did not use cognitive variables, Maltais *et al.* [33] reported the mean baseline FI to be 0.17 (SD 0.10), with 49% (131 participants) having a diagnosis of MCI. They did not report the prevalence of baseline frailty in this cohort, specifically in preclinical AD. This differs from our study, which had a larger sample size of 4486 participants who had a CDR of 0 and a mean FI of 0.2 (SD 0.1). Confirmation of our findings of the prevalence of frailty in preclinical AD should occur using other preclinical AD clinical trials such as AHEAD 3–45 [34] and TRAILBLAZER-ALZ3 [35], and observational AD studies where Aβ biomarkers (PET, cerebrospinal fluid, blood-based biomarkers) are available, and a diagnosis of preclinical AD can be made such as the Alzheimer’s Disease Neuroimaging Initiative [36] or the Australian Imaging, Biomarkers and Lifestyle study [37].

In a cognitively unimpaired cohort, our study found that Aβ+ participants were more likely to be frail compared to Aβ– participants, adjusted for age, sex, education and APOE ε4 carrier status. This finding varies from the cross-sectional reporting of Maltais *et al.* [33] in the MAPT study, who found no association between mean cortical Aβ load (SUVR 1.17) and frailty, but found an association between Aβ in the anterior putamen, posterior putamen and praecuneus regions with frailty at baseline. In a study in South Korea of 48 participants with MCI who had an amyloid PET and frailty assessed by Fried’s phenotype criteria, Yoon *et al.* [38] reported an association between weakness and Aβ accumulation, and gait speed and Aβ accumulation in the temporal cortex, parietal cortex, praecuneus/posterior cingulate cortex, and the basal ganglia. Our study examined participants who were cognitively intact with a larger sample size. Future studies could examine if Aβ load in specific brain regions are associated with frailty in preclinical AD.

Frail participants had lower PACC scores on average compared to non-frail participants after adjusting for age, sex, education and amyloid status. These findings are independent of amyloid status in cognitively unimpaired participants, where Aβ+ participants had lower PACC scores on average than Aβ– participants [15]. Frailty did not moderate the relationship between amyloid status and cognition. Frailty is an important predictor of future cognitive decline in cognitively unimpaired people, as increased frailty severity increases the risk of MCI/dementia, independent of APOE ε4 carrier status [9]. The relationships between frailty and cognition [39, 40], and between amyloid and cognition [15, 41] have already been quantified in previous studies, but to our knowledge, there have been no studies that have looked at the independent association after adjusting for frailty’s correlation with amyloid status. It should be noted that there was a statistical significance between APOE ε4 carrier status between frail and non-frail participants in the FI-CVR model, but no statistical significance in the FI-Full model despite similar proportions between the two FI models.

Future studies are required to understand the relationship between frailty, amyloid burden and cognition and the underlying mechanisms in preclinical AD clinical trial and observational AD cohorts. Longitudinal outcomes examining if frailty at baseline in preclinical AD influences cognition, functional status, safety outcomes and clinical trial attrition are warranted. Given frailty severity increases and accelerates 4–9 years prior to dementia [8], temporal relationships that should be explored include the changes in frailty in relation to amyloid, tau, neuroinflammation, neurodegeneration and synaptic dysfunction biomarkers in preclinical AD to progression to MCI/dementia. The MAPT study showed no significant associations between plasma Aβ 42/40 and incident frailty [42] or Aβ load and incident frailty [33]. The larger sample size and long duration (4.5 years) of the A4 study provide an opportunity to explore these relationships further. Future studies exploring the relationship between frailty, amyloid burden and cognition should also be performed in cognitively impaired cohorts such MCI and mild AD given the emergence of amyloid-targeting therapies in this group and whether interventions targeting frailty in people with MCI and mild AD to reduce cognition decline would be of benefit.

The strengths of our study were the large sample size, and that it was multi-site and multinational. The limitations of our study were that the A4 study was a selective clinical trial cohort that was predominantly Caucasian, well-educated and excluded people who had unstable medical conditions. This affects the generalizability of our findings. Further studies are required in diverse populations. Another limitation was that this was a cross-sectional analysis. Longitudinal analyses are required to understand the temporal nature of our findings.

Overall, this study shows that frailty was common in a cohort of cognitively unimpaired older adults. Frailty was associated with measurable cognitive changes, independent of amyloid status. Our findings are consistent with emerging literature that demonstrate the importance of frailty to future cognitive dysfunction and dementia. Evaluating the association of frailty within longitudinal outcomes in this cohort will be of interest.

## Supplementary Material

aa-25-1502-File002_afaf378

## Research Data Transparency and Availability

The A4 and LEARN Studies were coordinated by ATRI at the University of Southern California, and the data are made available under the auspices of Alzheimer’s Clinical Trial Consortium through the Global Research & Imaging Platform (GRIP).

## Notes

**Clinical trials registration number:** NCT02008357 (ClinicalTrials.gov)
