# Supplementary material for: Prevalence of frailty and its association with cognition in preclinical Alzheimer’s disease: a cross-sectional analysis of baseline data from the A4 study
Source: Age Ageing. 2026 Jan 22;55(1):afaf378. doi: 10.1093/ageing/afaf378 (PMC12825300; doi:10.1093/ageing/afaf378)
Supplement: aa-25-1502-File002_afaf378 [file aa-25-1502-file002_afaf378.docx]

**Manuscript Title: Prevalence of frailty and its association with cognition in preclinical Alzheimer’s disease: A cross-sectional analysis of baseline data from the A4 study**

# **SUPPLEMENTS**

**Appendix Table 1.** Frailty Index (51 health deficits in FI-Full; 36 health deficits in FI-CVR)

| Memory loss* | Difficulty with complex activities* | Skin problems on physical exam |
| --- | --- | --- |
| Repetition* | Poor sleep | Abnormal cardiovascular exam |
| Misplacing objects* | Cardiovascular disease | Abnormal musculoskeletal exam |
| Difficulty with dates* | Musculoskeletal disease | Abnormal HEENT exam |
| Difficulty with recall* | Head, Eyes, Ears, Nose, Throat (HEENT) | Swelling on physical exam |
| Difficulty driving* | Major Surgical Procedures | Abnormal respiratory exam |
| Help managing finances* | Respiratory disease | Abnormal gastrointestinal exam |
| Social isolation | Gastrointestinal disease | Impaired gait |
| Visuospatial problems* | Psychiatric disease | Weakness on neurological exam |
| Difficulty with appliances/devices* | Neurologic (other than AD) disease | Impaired sensorium* |
| Difficulty with transport | Renal-Genitourinary disease | Tremor on neurological exam |
| Difficulty with appliances/home  maintenance | Dermatologic-Connective Tissue disease | Cerebellar dysfunction on neurological exam |
| Help with laundry | Endocrine-Metabolic disease | Abnormal cranial nerves on neurological exam |
| Help with shopping | Malignancy | Abnormal body mass index |
| Help with meals | Hematopoietic-Lymphatic disease | Abnormal blood pressure |
| Help to use the telephone* | Hepatic disease | Anxiety (As per STAI)* |
| Difficulty managing medications* | Other medical conditions | Depression (As per GDS)* |

* Health deficits removed from the calculation of (FI-CVR) excluding possible confounding variables

STAI = State-Trait Anxiety Inventory

GDS = Geriatric Depression Scale

The item psychiatric disease would include psychiatric medical co-morbidities such as depression and anxiety, which was determined by the clinician reviewing a participant’s medical history. The items anxiety and depression were determined using questionnaires (Anxiety - STAI; depression -GDS).

**Appendix Table 2** Scoring and prevalence of variables in frailty index (FI)

| **Variable** | **A4 Code** | **FI deficit coding** | **Prevalence** |
| --- | --- | --- | --- |
| Memory loss* | 1=Yes  0=No  2=Maybe | 1=1  2=0.5  0=0 | 23.72% |
| Repetition* | 1=Yes  0=No  2=Maybe | 1=1  2=0.5  0=0 | 10.95% |
| Misplacing objects* | 1=Yes  0=No  2=Maybe | 1=1  2=0.5  0=0 | 32.03% |
| Difficulty with dates* | 1=Yes  0=No  2=Maybe | 1=1  2=0.5  0=0 | 13.91% |
| Difficulty with recall * | 1=Yes  0=No  2=Maybe | 1=1  2=0.5  0=0 | 62.53% |
| Difficulty driving * | 1=Yes  0=No  2=Maybe  -2=Does not apply | 1=1  2=0.5  0=0  -2=0 | 13.71% |
| Help managing finances* | 1=Yes  0=No  2=Maybe  -2=Does not apply | 1=1  2=0.5  0=0  -2=0 | 4.97% |
| Social isolation | 1=Yes  0=No  2=Maybe | 1=1  2=0.5  0=0 | 11.73% |
| Visuospatial problems* | 1=Yes  0=No  2=Maybe | 1=1  2=0.5  0=0 | 13.51% |
| Difficulty with appliances/devices* | 1=Yes  0=No  2=Maybe | 1=1  2=0.5  0=0 | 3.01% |
| Difficulty with transport | 3=As well as usual, with no difficulty  2=With a little  Difficulty  1=With a lot of  Difficulty  0=You did not do this activity | 0=1  1=1  2=0.5  3=0 | 3.52% |

| Difficulty with appliances/home  maintenance | 3=As well as usual, with no difficulty  2=With a little  Difficulty  1=With a lot of  Difficulty  0=You did not do this activity | 0=1  1=1  2=0.5  3=0 | 1.07% |
| --- | --- | --- | --- |
| Help with laundry | 3=As well as usual, with no difficulty  2=With a little  Difficulty  1=With a lot of  Difficulty  0=You did not do this activity | 0=1  1=1  2=0.5  3=0 | 12.8% |
| Help with shopping | 3=As well as usual, with no difficulty  2=With a little  Difficulty  1=With a lot of  Difficulty  0=You did not do this activity | 0=1  1=1  2=0.5  3=0 | 0.67% |
| Help with meals | 3=As well as usual, with no difficulty  2=With a little  Difficulty  1=With a lot of  Difficulty  0=You did not do this activity | 0=1  1=1  2=0.5  3=0 | 2.52% |
| Help to use the telephone* | 3=As well as usual, with no difficulty  2=With a little  Difficulty  1=With a lot of  Difficulty  0=You did not do this activity | 0=1  1=1  2=0.5  3=0 | 1.18% |
| Difficulty managing medications* | 3=As well as usual, with no difficulty  2=With a little  Difficulty  1=With a lot of  Difficulty  0=You did not do this activity | 0=1  1=1  2=0.5  3=0 | 6.58% |

| Difficulty with complex activities* | 3=As well as usual, with no difficulty  2=With a little  Difficulty  1=With a lot of  Difficulty  0=You did not do this activity | 0=1  1=1  2=0.5  3=0 | 3.52% |
| --- | --- | --- | --- |
| Poor sleep | 0 - 15 | 0-5 = 1  10-15=1  6-9=0 | 7.33% |
| Cardiovascular disease |  | Has condition=1  Does not=0 | 65.8% |
| Musculoskeletal disease |  | Has condition=1  Does not=0 | 70.15% |
| Head, Eyes, Ears, Nose, Throat (HEENT) |  | Has condition=1  Does not=0 | 55.24% |
| Major Surgical Procedures |  | Has condition=1  Does not=0 | 73.81% |
| Other medical conditions |  | Has condition=1  Does not=0 | 27.53% |
| Respiratory disease |  | Has condition=1  Does not=0 | 22.29% |
| Gastrointestinal disease |  | Has condition=1  Does not=0 | 47.48% |
| Psychiatric disease |  | Has condition=1  Does not=0 | 28.96% |
| Neurologic (other than AD) disease |  | Has condition=1  Does not=0 | 30.83% |
| Renal-Genitourinary disease |  | Has condition=1  Does not=0 | 40.79% |
| Dermatologic-Connective Tissue disease |  | Has condition=1  Does not=0 | 32.28% |
| Endocrine-Metabolic disease |  | Has condition=1  Does not=0 | 52.25% |
| Malignancy |  | Has condition=1  Does not=0 | 12.04% |
| Hematopoietic-Lymphatic disease |  | Has condition=1  Does not=0 | 9.65% |
| Hepatic disease |  | Has condition=1  Does not=0 | 3.52% |
| Abnormal HEENT exam | 1=Normal 2=Abnormal | 2=1  1=0 | 17.37% |
| Abnormal cardiovascular exam | 1=Normal 2=Abnormal | 2=1  1=0 | 9.41% |
| Abnormal respiratory exam | 1=Normal 2=Abnormal | 2=1  1=0 | 1.09% |
| Abnormal gastrointestinal exam | 1=Normal 2=Abnormal | 2=1  1=0 | 7.02% |
| Abnormal musculoskeletal exam | 1=Normal 2=Abnormal | 2=1  1=0 | 14.38% |
| Swelling on physical exam | 1=Normal 2=Abnormal | 2=1  1=0 | 9.41% |
| Skin problems on physical exam | 1=Normal 2=Abnormal | 2=1  1=0 | 20.4% |
| Impaired gait | 1=Normal 2=Abnormal | 2=1  1=0 | 9.76% |
| Weakness on neurological exam | 1=Normal 2=Abnormal | 2=1  1=0 | 2.96% |
| Impaired sensorium* | 1=Normal 2=Abnormal | 2=1  1=0 | 10.16% |
| Tremor on neurological exam | 1=Normal 2=Abnormal | 2=1  1=0 | 9.09% |
| Cerebellar dysfunction on neurological exam | 1=Normal 2=Abnormal | 2=1  1=0 | 2.9% |
| Abnormal cranial nerves on neurological exam | 1=Normal 2=Abnormal | 2=1  1=0 | 5.17% |
| Abnormal body mass index (BMI) |  | BMI <20=1  BMI >30=1  BMI 20-30=0 | 28.38% |
| Hypertension | Systolic blood pressure (SBP) 30 - 180 | SBP <90 = 1  SBP >140=1  SBP 90-140=0 | 37.2% |
| Anxiety* |  | STAI score >19=1 STAI score 17-19=0.5 STAI score <17=0 | 3.48% |
| Depression* |  | GDS score >10=1  GDS score 6-10=0.5  GDS score <6=0 | 2.05% |

STAI = State-Trait Anxiety Inventory

GDS = Geriatric Depression Scale

Help with shopping was excluded from the frailty index because the prevalence is <1%.

* Health deficits removed from the calculation of (FI-CVR) excluding possible confounding variables

The item psychiatric disease would include psychiatric medical co-morbidities such as depression and anxiety, which was determined by the clinician reviewing a participant’s medical history. The items anxiety and depression were determined using questionnaires (Anxiety - STAI; depression - GDS).
